# The Potential of Olive Leaf Extract as a Functional Ingredient in Yoghurt Production: The Effects on Fermentation, Rheology, Sensory, and Antioxidant Properties of Cow Milk Yoghurt

**DOI:** 10.3390/foods11050701

**Published:** 2022-02-26

**Authors:** Irena Barukčić, Katarina Filipan, Katarina Lisak Jakopović, Rajka Božanić, Marijana Blažić, Maja Repajić

**Affiliations:** 1 Department of Food Engineering, Faculty of Food Technology and Biotechnology, University of Zagreb, Pierottijeva 6, 10000 Zagreb, Croatia; ibarukcic@pbf.hr (I.B.); katarina.filipan@gmail.com (K.F.); rbozan@pbf.hr (R.B.); maja.repajic@pbf.unizg.hr (M.R.); 2 Department of Food Technology, Karlovac University of Applied Sciences, Trg J.J. Strossmayera 9, 47000 Karlovac, Croatia; marijana.blazic@vuka.hr; 3 Gastronomy Department, Aspira University College, Mike Tripala 6, 21000 Split, Croatia

**Keywords:** yoghurt, antioxidant activity, olive leaf extract, phenolic compounds, sensory properties, syneresis, viscosity, microbiological quality

## Abstract

Background: Yoghurt has been traditionally consumed for its high nutritional value and health-promoting benefits. The addition of plant extracts as a source of phenolic compounds and bio-flavonoids has attracted much attention recently since milk and dairy products are deficient in these health-protecting components. Accordingly, olive leaf extract (OLE) has been considered due to the presence of bioactive compounds, primarily polyphenols. Thus, the aim of this research was to investigate the possibility of adding OLE into cow milk yoghurt as a potential functional ingredient. Methods: Yoghurts enriched with OLE (1.5, 3, and 5% *v*/*v*) were produced and compared with yoghurt without OLE. In all samples acidity, viscosity, colour, syneresis, water holding capacity (WHC), microbiological parameters, sensory properties, total phenols, and antioxidant activity (DPPH and FRAP methods) were determined. Results: The addition of OLE resulted in shorter fermentation and lower pH, but it had no adverse effect on the viability of yoghurt starter bacteria. OLE-enriched yoghurts showed increased syneresis, higher total phenols content, and antioxidant activity, while WHC and viscosity decreased. Sensory properties were slightly poorer for yoghurts containing higher OLE concentrations. Considering all of the obtained results, the addition of 1.5% OLE appeared to be optimal.

## 1. Introduction

Modern consumers are increasingly aware of the important role that a well-balanced daily diet plays in preventing the occurrence of human health disorders. They look for benefits beyond nutrition and choose foods that provide wellness, convenience, and improved health [1]. Thus, regular consumption of functional foods containing specific components with a positive impact on human health has become unquestionable, and consumers are willing to pay up to 10–50% price premium for nutritionally enhanced products [2]. Fermented dairy products such as yoghurt or kefir have traditionally been considered foods of high nutritional value and numerous health-promoting benefits, which makes them one of the most frequently consumed functional foods in general. In this context, the development of functional dairy products is a very important area of expansion in the dairy industry, constituting one of the largest sectors in the global market of functional foods. The development of novel functional dairy products usually involves the addition of probiotic microorganisms, oligosaccharides, prebiotic fibres, conjugated linoleic acid, omega-3 fatty acids, phytosterols, minerals, vitamins, and bioactive peptides [1,3]. Most recently, the addition of plant extracts as a source of functional ingredients such as phenolic compounds and bio-flavonoids has gained considerable attention, since milk and dairy products are deficient in such valuable, health-protecting components [4]. Among numerous plant extracts that have been under the loop of investigations, olive leaf extract (OLE) has been recognised by the European Medical Agency as an official herbal preparation displaying a broad variety of health beneficial properties [5]. Olive polyphenols are bioactive components responsible for such health-protecting properties, whereby oleuropein is the most abundant one, constituting approximately 75% of all olive polyphenols [6]. In vivo and in vitro studies have indicated that oleuropein exhibits various biological activities such as antimicrobial, antioxidant, antihypertensive, anticoagulant, hypolipidemic, and antitumor properties [6,7,8,9]. Olive leaves account for up to 10% of the total olive mass to be processed, which globally represents a huge amount of food waste remaining after olive oil production [9]. Considering these data, except for improving the therapeutic value of dairy products, the addition of OLE could also contribute to enhancing sustainability in this sector of the food industry. Some authors have already studied the possibilities of adding OLE to yoghurt, in order to examine the options of supplementation (liquid, concentrated, powdered, encapsulated, pure oleuropein), as well as to increase its shelf life and/or functionality [6,8,9,10]. In these studies, the used extracts were usually obtained by boiling in hot water [6,10] or extracted from organic solvents such as ethanol, subsequently subjecting them to further processing operations such as freeze-drying, evaporation, or encapsulation [8,10,11], to obtain a higher concentration of active compounds to be added into yoghurt. From the perspective of developing new products, employment of so many intermediate steps might not simply appear unpractical but also expensive for large-scale applications. Additionally, due to a large pool of different data, there are still no uniform guidelines for levels of supplementation of yoghurt by OLE which could be applied in further research in developing this category of functional dairy products. In line with this aspect is also the fact that the so-far studied effects of OLE addition to yoghurt cannot be used as general findings because of distinctions in the type of the used supplement. Taking all of these facts into consideration, this study was performed in continuance to previous research focused on finding optimal extraction method for obtaining OLE [12,13] ready to use for yoghurt supplementation, without any intermediate steps of extract preparation. The objectives of this study were to investigate the effect of adding OLE on the fermentation process and viability of lactic acid bacteria contained in yoghurt culture, rheological, sensory, and antioxidant properties of yoghurt, as well as to determine the most appropriate levels of supplementation based on the obtained data.

## 2. Materials and Methods

### 2.1. OLE Production

Olive leaves (*Olea europaea* L., variety Oblica) used for the extraction were collected during the summer of 2019 in the area of Zadar county (Croatia), air-dried, and milled. OLE was produced by accelerated solvent extraction (ASE) in static mode using Dionex^TM^ ASE^TM^ 350 Accelerated Solvent Extractor (Thermo Scientific, Waltham, MA, USA), with distilled water as an extraction solvent. ASE was performed according to the procedure previously described by Dobrinčić et al. [12] but slightly modified according to Repajić et al. [13]. Briefly, extraction cells (34 mL) fitted with two cellulose filters (Dionex™ 350/150 Extraction Cell Filters, Thermo Fisher Scientific Inc., Waltham, MA, USA) at the bottom were filled with a mixture of samples (2.5 g) and diatomaceous earth (0.5 g). Extraction was carried out under the following conditions: temperature 100 °C, static extraction time 5 min, 3 extraction cycles, and pressure 10.34 MPa. After extraction, cells were flushed with solvent (50%) and purged with nitrogen (30 s). The obtained extracts contained on average 69.23 ± 0.87 mg g^−1^ oleuropein, 0.90 ± 0.07 mg g^−1^ hydroxytyrosol, 0.39 ± 0.02 mg g^−1^ chlorogenic acid, 0.27 ± 0.01 mg g^−1^ caffeic acid, 0.43 ± 0.02 mg g^−1^ verbascoside, and 2.37 ± 0.05 mg g^−1^ rutin, with the total sum of polyphenolic components of 73.59 ± 0.93 mg g^−1^, as previously published by Dobrinčić et al. [12]. The extracts were stored at −18 °C until further use.

### 2.2. Yoghurt Production

Pasteurised and homogenised cow milk containing on average 3.2 g milk fat, 4.6 g carbohydrates, and 3.3 g proteins per 100 mL (Dukat Ltd., Zagreb, Croatia) was used for solid yoghurt production. Milk was preheated to approximately 43 °C, divided into smaller portions anticipated for each sample batch, and supplemented by adding 1.5, 3, and 5% (*v*/*v*) of OLE. The control sample was not supplemented with OLE. Subsequently, milk samples were inoculated with thermophilic lyophilised yoghurt culture YoMix 10 DCU (Danisco-DuPont, Wilmington, IL, USA), according to the manufacturer’s directions, and fermented until reaching pH 4.60. The fermentation process was monitored by measuring the pH value of samples in predefined time intervals and stopped by rapid cooling in an ice-cold water bath. The produced samples were cool stored at +4 °C for 35 days and subjected to analyses in 7-day intervals, i.e., on 1st, 7th, 14th, 21st, 28th, and 35th day after production. For each storage time-period evaluation, three independent yogurts were analysed for each level of supplementation.

### 2.3. Acidity Measurements

Active acidity (pH) of yoghurt samples was determined by a pH meter Multi 340i (WTW, Wellheim, Germany) and titratable acidity (% lactic acid), according to the modified method of Soxhlet Henkel [14], which consisted of using 20 g of fermented milk homogenised in 20 mL of sterile distilled water and further titrated with 0.1 M NaOH (Merck GmBH, Germany).

### 2.4. Syneresis, Water Holding Capacity, and Viscosity

Syneresis (S) and water holding capacity (WHC) were determined using a modified centrifugal method developed by Feng et al. [15] and a method described by Cardines et al. [13], respectively. Briefly, 20 g of yoghurt was weighed and centrifuged (Rotina 380R Hettich, Tuttlingen, Germany) at 5000 rpm for 10 min at 4 °C. The obtained supernatant was separated, and the residual precipitate was weighed. 

Syneresis index was determined according to Cardines et al. [16] and calculated following Equation (1):

S (%) = (weight (supernatant)/(weight (sample)) × 100 (1)

Similarly, WHC was calculated following Equation (2):

WHC (%) = (weight (drained gel)/(weight (sample)) × 100 (2)

A rotating rheometer Rheometric Scientific RM-180 (Rheometric Scientific, Inc., Piscataway, NJ, USA), with a cylindrical spindle (ϕ 30 mm, l = 45 mm), was used to determine rheological properties of yoghurt samples tempered at 20 °C. In this way, shear stress (τ) and apparent viscosity (µ in Pa s) were measured at shear rates ranging between 100 and 1290 m s^−1^. From the dependency of the logarithm of shear rate and the logarithm of the shear stress, a linear regression equation was obtained which was applied for determining the consistency coefficient K (Pas^n^), flow index (n), and the regression coefficient (R^2^) for each sample following the Ostwald–de Waele model.

### 2.5. Microbiological Analyses

All yoghurt samples were analysed for viable counts of lactobacilli and streptococci during the entire storage period by the pour plate method. The initial solution was prepared by diluting 20 g of yoghurt in 180 mL of sterile physiological solution (sodium chloride 0.9% (normal saline), USP, Sterile Grade, Intermountain) [17]. The obtained suspension was used for the preparation of further dilutions. The viable count of *Lactobacillus* sp. was enumerated at the De Man, Rogosa, and Sharp (MRS) agar (43 °C/48 h), while M-17 agar (37 °C/48 h) was used for *Streptococcus* sp. enumeration (both Biolife, Milan, Italy).

### 2.6. Total Phenols Content

The total phenols content was determined in OLE and all yoghurt samples by Folin–Ciocalteu method, and the standard curve was plotted using 500 mg L^−1^ gallic acid (Sigma–Aldrich, St. Louis, MO, USA) as a stock solution [18]. Ultimately, the equation obtained from the standard curve was as follows:

(3) $$Y=0.0035\times X\;\left(R^{2}=0.9995\right)$$

OLE samples were 5-fold diluted with distilled water to obtain the results in the range of acceptability (absorbance up to 1). Prior measurements, as well as control and OLE-enriched yoghurt samples, were prepared according to Perna et al. [19], i.e., 20 g of yoghurt was centrifuged at 5000× *g* at 4 °C for 20 min. The obtained supernatant was filtered through Whatman no. 40 filter paper (Whatman International Ltd., Kent, UK), and the obtained filtrate was used for analysis.

### 2.7. Antioxidant Activity by Ferric Reducing Antioxidant Power (FRAP) and the DPPH Method

The ferric reducing antioxidant power (FRAP) assay was performed according to the procedure described by Benzie [20] and Benzie and Strain [21]. The samples were prepared as previously described. The standard curve was plotted using Trolox (Sigma–Aldrich, St. Louis, MO, USA) 2 mM stock solution, and the equation obtained from the standard curve was as follows:

(4) $$Y=0.0014\times X\;\left(R^{2}=0.9995\right)$$

The DPPH assay was performed according to the protocol described in detail by Tavakoli et al. [22].

The radical scavenging capacity was calculated from the following Equation:

Radical scavenging capacity (%) = ((A control − A sample)/A control) × 100(5)

### 2.8. Colour Determination

The colour of yoghurt samples was determined according to the CIElab system, using a CM-3500d spectrophotometer (Konica Minolta, Tokyo, Japan) with a D65 light source. The data were obtained in the SpectraMagic NX program. In order to determine whether the colour of samples supplemented by OLE differs from the control sample, the total colour difference (ΔE *) was calculated according to Equation (6) as follows:

ΔE * = √ ((L * − L *_ref_)^2^ + (a * − a *_ref_)^2^ + (b * − b *_ref_)^2^) (6)

where L *, a *, and b * refer to the test samples, and L *_ref_, a *_ref_, and b *_ref_ to the control sample [23].

### 2.9. Sensory Evaluation

Sensory evaluation of yoghurt samples was performed by a group of five specially trained panellists using a scoring system of weighted factors on a 20-point scale [24,25]. Yoghurt samples were cool stored at 4 °C from the point of production, until the point of sampling and evaluation. In a room designed according to ISO Standard 8589:2007 [26], samples were opened, encoded, divided into equal portions, and presented simultaneously to each of the five assessors. Samples were evaluated for overall appearance, colour, odour, consistency, syneresis appearance, and taste, whereby each attribute could have been rated with notes from 1 to 5. The average note of every attribute was multiplied with a predetermined weighting factor, resulting in a score for each attribute as follows: taste—10 scores; consistency—4 scores; odour and syneresis—2 scores each; overall appearance and colour—1 score each. By summarising the scores of each attribute, a final score for the particular sample was obtained. The maximum score that one sample could obtain was 20 [25].

### 2.10. Statistical Analysis

Each experiment was repeated in triplicate, and the obtained results were expressed as mean values ± standard deviations (SDs). Statistical analysis was performed using Statistica ver. 12.0 software (Statsoft Inc., Tulsa, OK, USA). After testing the normality and homoscedasticity of the data by Shapiro–Wilk and Levene tests, the data were analysed using ANOVA (syneresis, apparent viscosity, total phenols, and FRAP) or Kruskal–Wallis test (pH, acidity, WHC, viable counts of lactobacilli and streptococci, DPPH and all sensory attributes). Accordingly, means within groups were compared using Tukey’s HSD test or Kruskal–Wallis test. The significance level for all tests was set at *p* ≤ 0.05. Results of statistical analysis are presented as least squares (LS) means ± standard errors (SEs).

## 3. Results and Discussion

### 3.1. Acidity and Fermentation Time

The acidity of samples was monitored during the fermentation process in order to examine whether OLE affects the fermentation time. As presented in Table 1, it could be observed that OLE addition positively affected the fermentation process by reducing the fermentation time in all supplemented samples (OLE 1.5%, OLE 3%, and OLE 5%) in comparison with the control sample. The decrease in fermentation time was proportional to the increase in the added OLE. Thus, the average fermentation time of the control sample was 276 min, while the fermentation times of supplemented samples ranged from 240 min (OLE 3%) to 270 min (OLE 1.5%).

**Figure 1:**
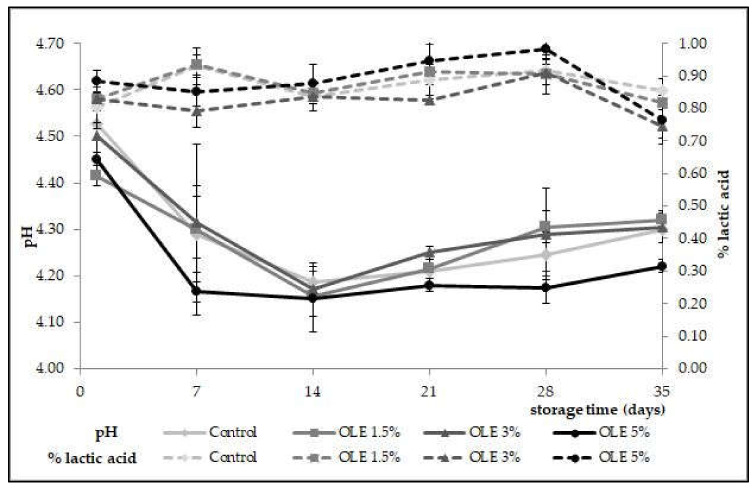
Changes in pH values and titratable acidity (% lactic acid) in yoghurt samples without (control) and with olive leaf extract addition (OLE 1.5%, OLE 3%, and OLE 5%) during 35 days of cold storage.

The acidity of the produced samples was monitored during 35 days of cold storage, as presented in Figure 1. Generally, pH values ranged from 4.22 (OLE 5%) to 4.31 (OLE 3% and control) with the grand mean of 4.28 (Table 2), while titratable acidity expressed as % lactic acid amounted between 0.82% (OLE 3%) and 0.88% (OLE 5%), with the grand mean of 0.87% (Table 2). The highest decline in acidity could be observed in all samples during 14 days of storage, whereby the supplemented samples (OLE 1.5%, OLE 3%, and OLE 5%) had lower pH values and higher concentrations (%) of lactic acid than the control sample. Such results might be explained by the remaining activity of the yoghurt culture which continued converting lactose to lactic acid but in a less intensive manner, due to low storage temperature. Higher rates of acidity generated in supplemented samples might indicate a promoting role of OLE in metabolic activities of lactobacilli and streptococci contained in yoghurt culture. After 14 days of cold storage, acidity increased slightly in all samples, with similar values obtained for OLE 1.5% and OLE 3% (Figure 1), reaching approximately 4.31 by the end of the storage period.

The control sample reached the same final value, although the values observed between the 14th and 35th day were slightly lower than OLE 1.5% and OLE 3%. Conversely, the OLE 5% sample had the lowest acidity values throughout the entire storage period, regardless of the sampling point. Additionally, during the last week of storage, an increase in the pH value was clearly accompanied by a decrease in % lactic acid (Figure 1). Statistical analysis showed that there was no significant difference (*p* > 0.05) in pH values and % lactic acid among samples with respect to OLE addition (Table 2). However, when considering the influence of the storage time on the pH value of the samples, significant differences (*p* < 0.001) could be observed among the 1st, 14th, 21st, and 28th days of storage.

The obtained results are in agreement with recent studies published by Kwon et al. [27] and Georgakouli et al. [28], respectively, which showed that the addition of plant extracts considerably enhances the production of lactic acid, which, in turn, leads to a faster decrease in pH and reaching the fermentation end point at pH 4.6. Similarly, Pourghboran et al. [10] and Marhamatizadeh et al. [29] also observed a positive correlation between the increased acidity and higher concentrations of added OLE and proposed that OLE addition promotes the growth of added starter strains responsible for acid formation during milk fermentation. Viable counts of Streptococcus thermophilus were slightly higher in OLE supplemented samples (Table 3) than the control sample, which might have affected the conversion rates of lactose into lactic acid, leading to the observed reduction in fermentation time (Table 1). In line with this are also the results for the acidity of yoghurt samples which was slightly higher for OLE-enriched samples than the control sample (Figure 1). The values obtained for pH (range 4.22–4.53) were consistent with the results previously published by Zoidou et al. [6,30], Cho et al. [8], and Pourghboran et al. [10], who measured pH in the range from 4.30 to 4.46 [6,8,30] and 4.30 to 4.50, respectively [10]; by contrast, titratable acidity (0.74–0.88% lactic acid) was slightly lower than the values measured by Pourghboran et al. [10] (0.93–1.01% lactic acid), Cho et al. [8] (0.88–0.96% lactic acid) and Tavakoli et al. [22] (0.89–1.08% lactic acid). In general, acidity values of OLE-enriched yoghurt samples produced in this study followed the same trends as those in previously published research [8,10,22,30], wherein the measured values decreased throughout the storage period, and no significant distinctions (*p* > 0.05) could be observed between the enriched and the control samples, regardless of the added OLE amount.

### 3.2. Syneresis, WHC, and Viscosity

**Figure 2:**
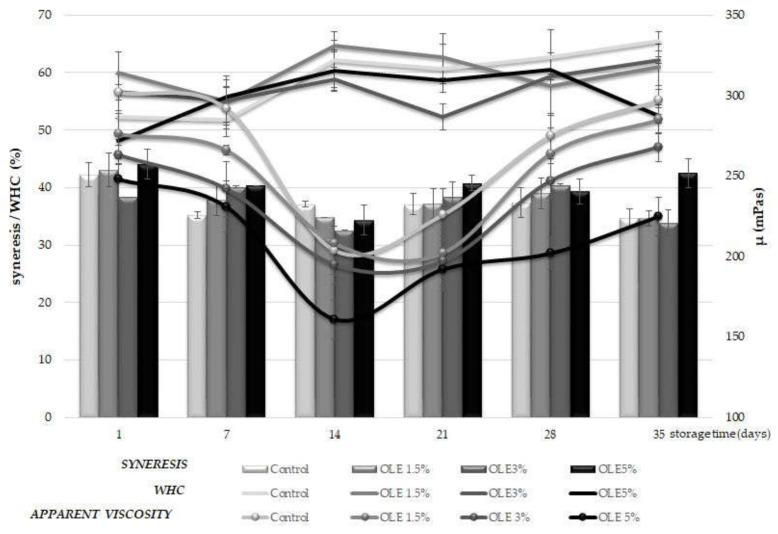
Average values of syneresis (S, %) and water holding capacity (WHC, %) in relation to average apparent viscosity (µ, m Pa s) at shear rate 100 s^−1^ of yoghurt samples without (control) and with olive leaf extract addition (OLE 1.5%, OLE 3%, and OLE 5) during 35 days of cold storage. S and WHC are placed onto primary *y*-axis, while apparent viscosity is on the secondary *y*-axis.

WHC is defined as the ability of yoghurt to hold all or part of its own water (or whey) [12]. Syneresis (S), on the other hand, is reflected through liquid (whey) separation on the surface of milk gel which is one of the most common defects in fermented milk products such as yoghurt. It can also be defined as the shrinkage of gel which occurs simultaneously with the expulsion of liquid or whey separation and is related to instability of the gel network, resulting in the loss of the ability to entrap all the serum phase [31,32]. The percentage of S and WHC through the monitoring period of 35 days for all treatments is presented in Figure 2.

The obtained values for syneresis were higher for OLE-enriched samples than the control sample during the entire storage period, except on the 14th and the 35th day, during which the highest values could be observed for the sample OLE 5% (Figure 2). The grand mean value of syneresis was calculated to be 38% (Table 2) which corresponded to approximately 7.68 mL per 20 g of yoghurt sample. Generally, there was a certain drop in syneresis values within the first 14 days of the storage period remaining afterward more or less at the same level or showing a slight increase, which was most obvious for sample OLE 5% on the 35^th^ day (Figure 2). Statistical analysis revealed no significant differences (*p* > 0.05) in syneresis among samples regarding the amount of added extract (Table 2). By contrast, considering the storage time, there were significant differences (*p* ≤ 0.05) in syneresis between samples on the 1st and 14th day, and the 1st and the 35th day, respectively (Table 2). 

WHC values showed opposite trends to syneresis during the entire storage period, which could be expected. More precisely, OLE-enriched samples showed higher WHC until the 28th day of storage, after which they were lower than the control sample (Figure 2), but again, statistical analysis revealed no significant difference (*p* > 0.05) among samples with respect to the addition of OLE (Table 2). The grand mean value of WHC was 58.0% (Table 2).

Considering the influence of storage time, WHC increased slightly until the end (Figure 2), but no significant differences could be observed (Table 2).

Although no significant differences in syneresis and WHC could be found among the produced yoghurt samples with respect to OLE addition (Table 2), it was evident that syneresis was somewhat higher in OLE-enriched samples, with the highest values determined in the sample OLE 5% (Figure 2). These results are similar to findings reported by Zoidou et al. [6,30], Tavakoli et al. [22], and Dönmez et al. [33], who also found increased syneresis rates of yoghurt samples enriched by adding extracts of olive phenols [6,22,30] or green tea and coffee powders [33], in comparison with the control sample, during 21 days of cold storage. Such results might be explained by the increased amount of added water caused by OLE supplementation and the insufficient ability of the milk protein network to incorporate it, as Tavakoli et al. [22] also suggested. When looking at the values of the same sample, there was a certain drop in syneresis and an increase in WHC during the first 14 days of storage (Figure 2). Despite not being significant (Table 2), this might indicate that there probably exist interactions between olive polyphenols and milk proteins (primarily casein), resulting in the formation of a protein network with smaller pores and greater water-binding capacities [34,35]. Therefore, the increase in acidity (Figure 1) might have promoted these processes since there are more binding sites on proteins at lower pH values due to the natural occurrence of dissociation [36,37]. The polyphenol–protein complexes formed during that period might have caused WHC and viscosity values to increase until the end of the storage period (Figure 2). This phenomenon could especially be noticed for sample OLE 1.5% which continuously showed higher WHC values than the control sample up to the 28th day of storage, indicating that this amount of OLE might be optimal to ensure previously suggested interactions between olive polyphenols and milk proteins.

Regarding the apparent viscosity of the samples, the highest values were recorded for the control sample at the 1st (302 ± 9 mPas) and the 35th day of storage (327 ± 5 mPas), respectively (Figure 2). The addition of OLE caused a decrease in viscosity, which was more or less proportional to the increase in the added OLE amount, so the lowest viscosity values were recorded in the sample OLE 5% on the 1st (248 ± 13 mPas), the 14th (161 ± 12 mPas), and the 35th day (225 ± 12 mPas), respectively (Figure 2). As could be expected, statistical analysis revealed a significant difference (*p* < 0.05) in apparent viscosity between the control sample and the sample OLE 5% with respect to the addition of extract (Table 2).

Overall, during the entire storage period, the highest viscosity was recorded for the control sample, but the values decreased with time. The highest decrease in viscosity was observed on the 14th day of storage (Figure 2), which also corresponded to the observed decrease in pH values (Figure 1) detected during the first 14 days. Such trends were also reported by Cho et al. [8] and El-Messery et al. [11], who also detected a decrease in viscosity of all samples during 14 days of cold storage. After that period, viscosity started to increase, reaching at the end almost identical values as on the first day for some samples (control, OLE 1.5%, and OLE 3%) (Figure 2). The only exception was again the OLE 5% sample which remained at the lowest apparent viscosity values throughout the entire storage period. Accordingly, significant differences (*p* < 0.001) among samples were determined between the 14th and the 28th day, and between the 14th and 35th day, respectively (Table 2). Along with previously discussed trends in syneresis and WHC values for all samples except OLE 5%, the obtained results might implicate that OLE supplementation with concentrations up to 3% (*v*/*v*) do not negatively influence the rheological behaviour of yoghurt and might lead to its improved viscosity of yoghurt. Similar conclusions were drawn by Zoidou et al. [30], who claimed that oleuropein supplementation of yoghurt resulted in improved viscosity and texture.

By definition, the *n*-value, known also as the flow index, represents the deviation from the Newtonian flow, being for shear-thinning fluids *n* < 1 and for Newtonian fluids *n* = 1 [38]. According to the obtained values for flow index (Table 3), all yoghurts belong to pseudoplastic non-Newton fluids, wherein the highest deviation from Newtonian behaviour could be observed for sample OLE 5%. This sample also showed the lowest consistency coefficients in comparison with all other samples during the entire storage period (Table 3) which was in good correspondence to the obtained values of apparent viscosity (Figure 2). The obtained n and K values were similar to those obtained by Domgala [39] for cow milk yoghurt; the authors examined rheological parameters of cow, goat, and sheep yoghurt on the 1st and the 14th day of storage. It could also be observed that the K value increased throughout the storage period for all samples, reaching the highest values at the end of the storage. Additionally, the control sample was characterised by the highest consistency coefficients, while the values observed for sample OLE 1.5% were the nearest and most similar to the control sample. Such trends corresponded well to trends in apparent viscosity and WHC (Figure 2), also indicating a thickening effect by the end of the storage period for all samples. Regression coefficients (R^2^) reveal the method’s accuracy, and they fitted relatively well to the Ostwald–de Waele model, as all values ranged above 0.900.

### 3.3. Microbiological Analyses

Viable counts of *Lactobacillus* sp. and *Streptococcus* sp. (log CFU mL^−1^) are presented in Table 3. In general, it could be observed that viable counts of both species constantly decreased in all samples during 35 days of cold storage. The highest values were observed in the control sample, while the addition of OLE caused a certain drop which was proportional to the added amount. *Lactobacillus* sp. showed lower viable cell counts in all OLE-enriched samples and a constant decrease for all samples, throughout the entire storage period, with the lowest counts determined for sample OLE 5%. In comparison with *Lactobacillus* sp. (grand mean value 7.32 log CFU mL^−1^), viable counts for *Streptococcus* sp. were considerably higher (grand mean value 9.50 log CFU mL^−1^), which might imply a certain inhibitory effect of OLE on lactobacilli. Statistical analysis also revealed no significant difference (*p* > 0.05) in viable counts of both species among yoghurt samples considering the addition of OLE, but there was a significant difference (*p* ≤ 0.05) between the first and the last day of storage for both examined species (Table 2).

Thus, OLE addition had no significant effect (*p* > 0.05) on the viability of bacterial strains of *Lactobacillus* sp. and *Streptococcus* sp. (Table 2), indicating that OLE, although being a strong antimicrobial agent, does not inhibit the growth of the used yoghurt starter culture. These findings are consistent with previously published results of Zoidou et al. [6,30] and Pourghorban et al. [10], who supplemented cow milk yoghurt with different concentrations of pure oleuropein and with OLE, respectively, and found no adverse influence on yoghurt microflora. However, it could be observed that the viability of *Lactobacillus* sp. was from the start lower at the highest level of supplementation (OLE 5%); it decreased during the storage period in a similar manner in all samples and dropped below the recommended value of 10^7^ CFU g^−1^ [40] after 21 days in the control and 28 days in OLE-enriched samples, respectively (Table 4). From the presented results (Table 4), it is also obvious that *Streptococcus thermophilus* was more stable than *Lactobacillus* sp. throughout the entire storage period and survived above the recommended level 10^7^ cfu g^−1^ until the 35th day of cold storage. Same trend was observed by Pourghorban et al. [10]. Marhamatizadeh et al. [29] previously suggested that OLE promotes the growth of *Lactobacillus acidophilus* and *Bifidobacterium bifidum* in milk and yoghurt. Although we did not encounter a significant difference (Table 2) between the control and OLE-enriched samples, results presented in Table 4 open the possibility of further research for using OLE as a functional ingredient for yoghurt supplementation in concentrations up to 3% (*v*/*v*), to maintain the viability of *Lactobacillus* sp. at recommended levels of 10^7^ CFU g^−1^ in yoghurt after the usually stated “best before date” of 21 days.

### 3.4. Total Phenols and Antioxidant Activity

**Figure 3:**
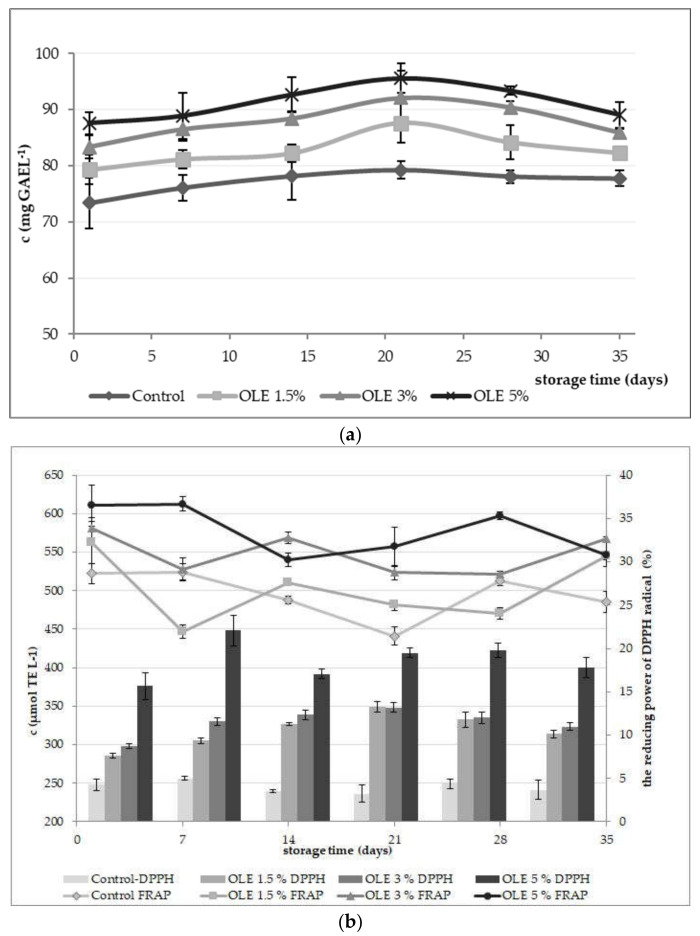
(**a**) The concentration of total phenols (c) expressed in mg of gallic acid equivalents (mg GAE L^−1^) in yogurt samples without (control) and with olive leaf extract addition (OLE 1.5%, OLE 3%, and OLE 5%) during 35 days of cold storage; (**b**) antioxidant activity measured by the DPPH assay expressed as the reducing power of DPPH radical (%) and the FRAP assay expressed as the concentration (c) of Trolox equivalents (µmol TE L^−1^) in yoghurt samples without (control) and with olive leaf extract addition (OLE 1.5%, OLE 3%, and OLE 5%) during 35 days of cold storage.

The content of total phenols in yoghurt samples ranged from approximately 80 to 100 mg GAE L^−1^ (Figure 3a), while OLE contained on average 990.71 mg GAE L^−1^. It could be observed that the content of total phenols increased with the amount of added OLE, with the highest values measured for sample OLE 5% (mean value 91.2 ± 1.7 mg GAE L^−1^). The same trend was also detected for values of antioxidant activity measured by DPPH and FRAP methods (Figure 3b). Accordingly, the highest value (612.86 µmol TE L^−1^) for antioxidant activity measured by the FRAP method was observed for sample OLE 5% which also showed the highest percentage (≥15%) of the DPPH reducing power (Figure 3a,b). The antioxidant activity of the OLE itself was determined by the FRAP method and amounted to 3457.14 µmol TE L^−1^.

Statistical analysis revealed significant differences (*p* < 0.001) between values of the reducing power measured by DDPH determined in the control sample and in all OLE-enriched samples (Table 2). Considering the content of total phenols and the antioxidant activity measured by FRAP, the control sample was statistically different (*p* < 0.001) from OLE 3% and OLE 5% samples (Table 2).

**Figure 4:**
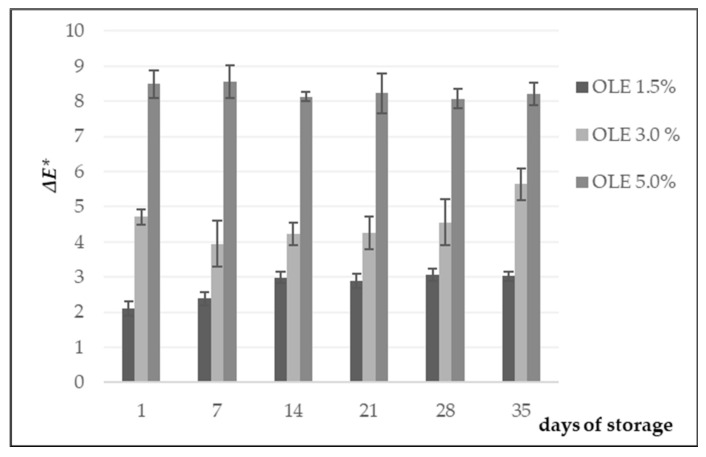
Total colour difference (ΔE *) of yoghurt samples enriched with different amounts of olive leaf extract (OLE 1.5%, OLE 3%, and OLE 5%).

When observing the total phenols amount with respect to the influence of the storage time, there was a certain increase during the first 21 days of storage, and significant differences (*p* < 0.001) were observed between the 1st and the 21st day, and the 1st and 28th day (Table 2), respectively. Considering the reducing power by the DPPH method, no significant changes (*p* = 0.680) were detected with respect to the storage time, while the antioxidant activity by the FRAP method was significantly different (*p* < 0.001) practically throughout the entire storage period, from the 1st until the 28th day (Table 2). The content of total phenols and the values of antioxidant activity (Figure 4) demonstrated that the supplementation of yoghurt with OLE represents an efficient pattern for enhancing its antioxidant capacity and biological value. Very similar results were obtained by Cho et al. [8], who added OLE at levels between 0.1% and 0.4% (*v*/*v*) into cow yoghurt and detected an increase in total phenol content in all OLE-supplemented samples, which was proportional to the added extract amount. Pourghorban et al. [10] also enriched yoghurt with olive leaf extract pre- and post-fermentation and also found it to increase the total phenol concentration. Oh et al. [41] measured total phenols concentration in cow milk yoghurt between 50 and 60 mg GAE L^−1^, while Vázquez et al. [42] found it to be 49 mg GAE L^−1^, which was lower than the results in the present study for the control sample (70–80 mg GAE L^−1^). As Chávez-Servín et al. [43] previously explained, phenols present in milk originate mainly from feed and are affected by factors such as season. Grazing feeding systems result in much higher phenols concentrations in milk, especially during hot and dry seasons. Since the milk used for yoghurt production was of commercial nature, one can only assume that milking cows were most probably not held on pasture and that the slightly higher concentrations of phenols are most probably related to a well-balanced feeding practice. Moreover, it could be possible that some milk components such as proteins, amino acids, unsaturated fatty acids, some vitamins (B1, B6, C, and folic acid), or different organic acids reacted with the Folin–Ciocalteu reagent, as Everette et al. [44] previously suggested. In order to overcome the limitations of this method, the antioxidant activity of all yoghurts was also determined (Figure 3b). It could be observed that the added plant extracts (OLE) significantly (*p* < 0.001) enhanced the antioxidant activity of yoghurt, especially at higher concentrations (OLE 3% and OLE 5%), which was similar to findings of some recent studies. Tomar et al. [45] examined the addition of various plant extracts (mint, basil, hibiscus) in concentrations (*v*/*v*) of 0.1%, 0.3%, and 0.5% to cow milk yoghurt and also found a linear increase in antioxidant activity measured by the DPPH method. Based on their findings, the activities for the control sample were almost identical (10–20% radical scavenging power) to the results in this study (Figure 3b). Our results for the DPPH method were more or less similar to the results of Pourghorban et al. [10] but lower in comparison to values obtained by Tavakoli et al. [22] and Cho et al. [8]. Despite the difference in the measured values, the observed trends indicated the same conclusion, i.e., OLE addition resulted in a significant increase in antioxidant activity of yoghurt. The same conclusions could be drawn from findings presented by Oh et al. [41]. Since it is well known that phenolic compounds exhibit antioxidant properties [46], the increase in antioxidant activity of OLE-enriched yoghurt samples in this study was most probably caused by polyphenolic compounds originating from olive leaves.

Accordingly, a certain decrease in antioxidant activity observed in all OLE yoghurt samples during the storage period (Figure 3b) was probably a result of the degradation of the phenolic compounds by the effect of starter bacteria [47] but also due to their interactions with proteins released by syneresis or peptides originating from proteolytic processes during storage, leading to the formation of polyphenol–protein complexes which do not enter DPPH or FRAP assay reactions.

### 3.5. Colour Determination and Sensory Evaluation

Colour is one of the most important attributes in dairy products; colour differences affect storage, shelf life, and colour deterioration of yoghurt, according to Coggins et al. [48]. The total colour difference (ΔE *) is important because it comprises all differences encountered among L *, a *, and b * colour values of the tested samples, whereby the control samples are taken into account. L * values represent lightness (0—black, medium values—greys, and 100—white), while values a * and b * can be presented as points in the ab coordinate system, on a colourful plane, where colours fluently pass from red (+a *) through yellow (+b *) to green (–a *) and blue (–b *) [31]. Values obtained for each OLE-enriched sample were compared with the reference sample (control) by calculating ΔE * [20] which is presented in Figure 4. According to Mokrzycki and Tatol [49], the ΔE * values (<1.0) indicated no difference between the control sample and test samples, or more precisely, OLE samples.

Considering the obtained results, it is evident that the difference in the colour of the samples increased with the amount of the added OLE. Sample OLE 1.5% showed a slightly noticeable difference up to the 28th day of storage, but after that period, it increased to a remarkable difference. Sample OLE 3% showed differences in comparison with the control sample during the entire storage period, while sample OLE 5% highly differed from the other samples (Figure 4). Statistical analysis revealed significant differences (*p* < 0.001) in ΔE * values between the control sample and OLE 3% and OLE 5% samples, considering the influence of OLE addition. However, no significant difference (*p* > 0.001) in ΔE * could be observed between the samples with respect to the storage time (Table 2).

**Figure 5:**
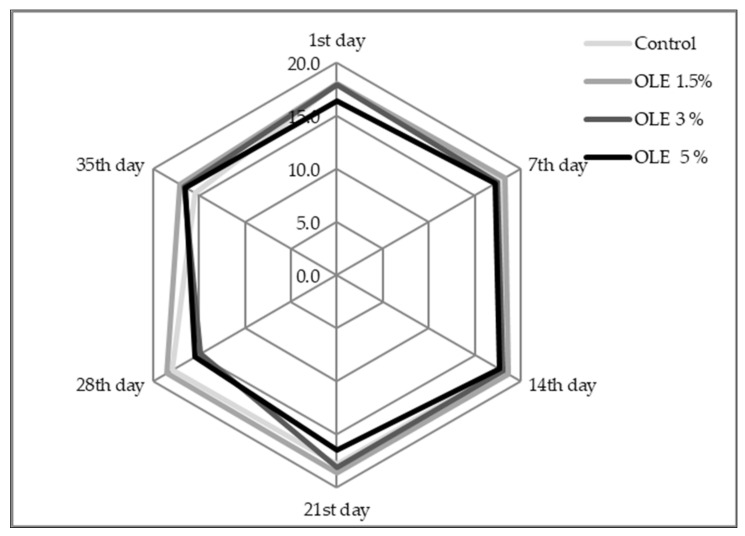
Total scores achieved by sensory evaluation of yoghurt samples without (control) and with the addition of olive leaf extract (OLE 1.5%, OLE 3%, and OLE 5%) during 35 days of cold storage.

Sensory evaluation of all samples showed that the OLE 1.5% sample achieved the highest scores during the entire storage period (Figure 5), followed by the control sample. Conversely, sample OLE 5% was evaluated with the lowest scores at all session times. After analysing the scores for each evaluated attribute, it is evident that taste was the most variable one, receiving the lowest scores with respect to the maximum possible (data not shown). Statistical analysis confirmed these results since there was a significant difference (*p* = 0.001) in taste between the control sample and OLE 5% and OLE 1.5% samples (Table 4). These findings could be linked to comments of panellists who perceived redundant acidity at the beginning and in the middle of the storage period for the control sample, and near the end of the storage period for OLE-enriched samples. Further, manifestations such as bitterness, strange (untypical), and herbal taste were also observed, especially at the end of the storage period. Thus, these observations were supported by statistical results which showed a significant difference (*p* ≤ 0.05) between samples on the 14th and the last (35th) day of storage (Table 4). Moreover, it is also worth mentioning that the yellowish colour of enriched samples was more evident in sample OLE 5% and towards the end of the storage period, as well as an untypical strange odour. This was the reason for the lower total scores of sample OLE 5% during the storage period (Figure 5).

Significant differences (*p* = 0.045) were detected in sensory-evaluated colour with respect to the addition of OLE between sample OLE 5% and all other samples, which corresponded to instrumental colour measurements (Table 2). Still, storage time did not show a significant influence (*p* = 0.067) on this attribute (Table 5). Considering the total score, the best-rated sample besides the control sample was OLE 1.5%, while sample OLE 5% achieved the lowest grades (Figure 5).

As presented, major differences among the control sample and OLE-enriched samples were detected in colour, odour, and taste (Table 5), whereby the higher the amount of added extract was, the more intense distinctions could be observed. Members of the evaluation panel often described sample OLE 5% as untypical, having off flavours reminding of green aromas and moulds and having an unappealing green colour and odour. Very often, comments regarding poorer consistency and odour, as well as slightly bitter taste, were also noted. Such comments reflected lower scores for OLE-enriched yoghurts, especially for samples OLE 3% and OLE 5%, during the storage period (Figure 5). Although instrumental measurement clearly resulted in large colour distinctions between OLE-enriched samples and the control sample (Table 2 and Figure 4), sensory evaluation was not that precise, and the panel noticed colour changes only for sample OLE 5% (Table 4). Such results corresponded to some previously published findings such as those of Dönmez et al. [33] and Tomar et al. [45], who detected considerable colour changes in yoghurt supplemented with green tea and coffee powder [33] or with mint, basil, or hibiscus extract [45], respectively. Cho et al. [8] Tavakoli et al. [22] also found that OLE addition negatively influenced colour, which was bright green, and the taste of yoghurt was often described as bitter and astringent. Similarly, Zoidou et al. [6,30] observed that the addition of OLE reflected a light green colour, a bitter taste, and a slight sharp after-taste of olive leaf, but it did not change the natural yogurt flavour. They also found no differences in consistency of both yoghurts, which was similar to sensory evaluation of this study (Table 5) but opposite to the instrumental results regarding viscosity (Figure 2), especially considering the influence of the storage period. Similar to our findings, Pourghorban et al. [10] also found that OLE supplementation to yoghurt in concentrations of 5 mg mL^−1^ was found unacceptable due to bitter flavour, undesirable colour, and texture. In general, it could be concluded that higher concentrations of OLE were not appreciated by the sensory panel with respect to the organoleptic characteristic of yoghurt, and the sample OLE 1.5% was perceived as the most desired, i.e., the most similar to the control sample (Figure 4).

## 4. Conclusions

The results of this study showed that OLE could be an effective ingredient for improving the nutritional and health benefits of cow milk and yoghurt. OLE addition caused a slight reduction in fermentation time (up to 0.5 h) probably by stimulating starter culture to faster acid production. Accordingly, OLE-enriched yoghurts showed lower pH values and higher syneresis than the control sample throughout the entire storage period, especially samples containing 3% and 5% of OLE. The utilisation of OLE in yoghurt had no significant effect on the viability of *Lactobacillus* sp. and *Streptococcus* sp., confirming that OLE, although being a strong antimicrobial agent, does not inhibit the growth of the used yoghurt starter culture. However, it could be observed that levels of supplementation 1.5% and 3% (*v*/*v*) were more appropriate for maintaining lactobacilli counts at recommended values of 10^7^ CFU g^−1^ up to 28 days of cold storage. The increased content of total phenols, as well as the values of antioxidant activity of the enriched yoghurts, demonstrated that the supplementation of yoghurt with OLE represents an efficient pattern for enhancing its antioxidant capacity and health-protecting properties. Sensory analysis revealed major differences among the control sample and OLE-enriched samples in colour, odour, and taste, whereby the higher the amount of added extract was, the more intense distinctions could be observed. Rheological parameters revealed that higher levels of supplementation (>1.5%) resulted in reduced apparent viscosity and coefficients of consistency. Finally, OLE showed a high potential to become a new functional yoghurt ingredient, but further research is needed to optimise its dosage, in order to overcome the problems associated with enhanced syneresis and changes in sensory properties detected within this study, and to investigate the influence of the most appropriate levels of supplementation (1.5%, 3%) on the shelf life of yoghurt.

## Figures and Tables

**Table 1:** Average pH values measured during fermentation in different yoghurt samples (control, OLE 1.5%, OLE 3%, and OLE 5%).

Time (min)	pH Value			
	Control	OLE 1.5%	OLE 3%	OLE 5%
0	6.64 ± 0.11	6.63 ± 0.10	6.64 ± 0.12	6.73 ± 0.11
60	6.56 ± 0.12	6.55 ± 0.09	6.52 ± 0.10	6.60 ± 0.11
120	6.25 ± 0.08	6.23 ± 0.07	6.12 ± 0.10	6.19 ± 0.06
180	5.42 ± 0.07	5.17 ± 0.10	5.09 ± 0.05	5.22 ± 0.09
210	5.28 ± 0.10	4.80 ± 0.09	4.88 ± 0.05	5.00 ± 0.07
240	4.91 ± 0.09	4.78 ± 0.07	**4.64 ± 0.02 (end)**	4.79 ± 0.09
261	4.73 ± 0.10	**4.63 ± 0.02 (end)**	/	4.71 ± 0.07
270	4.70 ± 0.07	/	/	**4.61 ± 0.02 (end)**
276	**4.64 ± 0.03 (end)**	/	/	/

Results are expressed as mean ± SD.

**Table 2:** Influence of extract content (%) and storage time on physical, chemical, and microbiological properties of yoghurts enriched with olive leaf extract.

Source of Variation	pH	Acidity (% Lactic Acid)	WHC (%)	Syneresis Index (%)	Apparent Viscosity (mPa s)	*Lactobacillus* sp. (log CFUmL^−1^)	*Streptococcus* sp. (log CFUmL^−1^)	Total Phenols (mg GAE L^−1^)	FRAP (µmol TE L^−1^)	DPPH (%)	ΔE *
**Extract content (%)**	*p* = 0.083	*p* = 0.473	*p* = 0.074	*p* = 0.206	*p* = 0.021 *	*p* = 0.369	*p* = 0.348	*p* < 0.001 *	*p* < 0.001 *	*p* < 0.001 *	*p* < 0.001 *
0	4.31 ± 0.04 ^a^	0.88 ± 0.03 ^a^	58.2 ± 2.3 ^a^	37.6 ± 0.9 ^a^	272.6 ± 20.1 ^b^	7.66 ± 0.67 ^a^	9.73 ± 0.30 ^a^	80.0 ± 1.8 ^a^	492.5 ± 8.4 ^a^	4.4 ± 0.5 ^a^	1.0 ± 0.2 ^a^
1.5	4.29 ± 0.03 ^a^	0.88 ± 0.03 ^a^	60.5 ± 1.7 ^a^	38.0 ± 1.3 ^a^	253.7 ± 13.5 ^ab^	7.13 ± 0.61 ^a^	9.70 ± 0.26 ^a^	82.3 ± 1.5 ^a^	502.6 ± 11.9 ^a^	11.5 ± 2.2 ^b^	2.1 ± 0.2 ^ab^
3	4.31 ± 0.03 ^a^	0.82 ± 0.02 ^a^	57.4 ± 1.2 ^a^	38.1 ± 1.0 ^a^	232.8 ± 14.2 ^ab^	7.86 ± 0.60 ^a^	9.47 ± 0.21 ^a^	88.4 ± 1.5 ^b^	548.4 ± 8.0 ^b^	12.8 ± 2.3 ^b^	4.0 ± 0.3 ^bc^
5	4.22 ± 0.03 ^a^	0.88 ± 0.04 ^a^	56.0 ± 1.5 ^a^	40.0 ± 1.2 ^a^	214.4 ± 16.8 ^a^	6.62 ± 0.61 ^a^	9.09 ± 0.07 ^a^	91.2 ± 1.7 ^b^	577.6 ± 9.6 ^b^	18.0 ± 2.0 ^b^	7.5 ± 0.5 ^c^
**Storage time (day)**	*p* < 0.001 *	*p* = 0.087	*p* = 0.054	*p* = 0.001 *	*p* < 0.001 *	*p* = 0.018 *	*p* = 0.006 *	*p* < 0.001 *	*p* = 0.002 *	*p* = 0.680	*p* = 0.994
1	4.48 ± 0.02 ^b^	0.82 ± 0.04 ^a^	54.8 ± 2.3 ^a^	42.4 ± 0.9 ^c^	248.6 ± 17.0 ^abc^	9.12 ± 0.78 ^b^	10.01 ± 0.32 ^b^	80.7 ± 1.5 ^a^	571.2 ± 10.5 ^b^	9.4 ± 1.5 ^a^	3.8 ± 1.2 ^a^
7	4.29 ± 0.04 ^ab^	0.86 ± 0.04 ^a^	52.7 ± 2.8 ^a^	38.1 ± 0.9 ^abc^	262.1 ± 11.6 ^bc^	7.97 ± 0.61 ^ab^	9.80 ± 0.31 ^ab^	83.6 ± 2.3 ^ab^	525.5 ± 20.2 ^a^	11.0 ± 4.1 ^a^	4.0 ± 1.2 ^a^
14	4.18 ± 0.02 ^a^	0.85 ± 0.01 ^a^	61.5 ± 1.1 ^a^	35.0 ± 1.1 ^a^	191.0 ± 18.1 ^a^	7.73 ± 0.79 ^ab^	9.54 ± 0.21 ^ab^	84.9 ± 2.4 ^abc^	524.6 ± 9.8 ^a^	11.0 ± 2.1 ^a^	3.2 ± 0.9 ^a^
21	4.22 ± 0.01 ^a^	0.93 ± 0.04 ^a^	59.2 ± 1.8 ^a^	38.4 ± 0.9 ^abc^	201.6 ± 11.9 ^ab^	7.13 ± 0.71 ^ab^	9.47 ± 0.38 ^ab^	91.6 ± 1.7 ^c^	503.2 ± 13.1 ^a^	10.5 ± 2.1 ^a^	3.5 ± 0.9 ^a^
28	4.25 ± 0.02 ^a^	0.94 ± 0.05 ^a^	59.3 ± 0.9 ^a^	40.4 ± 0.9 ^bc^	275.4 ± 26.4 ^c^	6.71 ± 0.70 ^ab^	9.21 ± 0.21 ^ab^	88.3 ± 2.0 ^bc^	525.6 ± 15.4 ^a^	12.1 ± 2.1 ^a^	3.7 ± 0.8 ^a^
35	4.29 ± 0.02 ^ab^	0.80 ± 0.02 ^a^	60.5 ± 2.0 ^a^	36.2 ± 1.7 ^ab^	281.5 ± 17.0 ^c^	5.26 ± 0.17 ^a^	8.96 ± 0.07 ^a^	83.8 ± 2.7 ^ab^	531.5 ± 11.3 ^ab^	16.1 ± 4.2 ^a^	3.6 ± 1.0 ^a^
**Grand mean**	4.28	0.87	58.0	38.4	243.4	7.32	9.50	85.5	530.3	11.7	3.6

WHC = water holding capacity. * *p* ≤ 0.05. Results are expressed as mean ± SE. Values with different superscript letters within column are statistically different at *p* ≤ 0.05.

**Table 3:** Parameters of rheological behaviour (flow index (*n*); consistency coefficient *K* (Pas^n^); coefficient of regression (*R^2^*)) according to Ostwald–de Waele model in the control sample and OLE-enriched yoghurt samples (OLE 1.5%, OLE 3%, and OLE 5%) during 35 days of cold storage.

Days of Storage	1	7	14	21	28	35
***Sample***	***Flow index (n)***					
***Control***	0.28 ± 0.04	0.23 ± 0.02	0.25 ± 0.02	0.24 ± 0.01	0.22 ± 0.01	0.23 ± 0.02
***OLE 1.5%***	0.28 ± 0.01	0.25 ± 0.04	0.26 ± 0.02	0.26 ± 0.03	0.24 ± 0.02	0.23 ± 0.02
***OLE 3%***	0.31 ± 0.02	0.28 ± 0.01	0.26 ± 0.03	0.24 ± 0.04	0.25 ± 0.03	0.22 ± 0.00
***OLE 5%***	0.34 ± 0.03	0.32 ± 0.01	0.29 ± 0.02	0.30 ± 0.04	0.32 ± 0.03	0.30 ± 0.01
	***Consistency coefficient (K)/Pas^n^***					
***Control***	4.15 ± 0.07	5.94 ± 0.04	5.65 ± 0.09	6.26 ± 0.10	6.96 ± 0.08	6.25 ± 0.11
***OLE 1.5%***	3.70 ± 0.11	4.11 ± 0.11	4.41 ± 0.08	4.03 ± 0.07	5.38 ± 0.06	5.79 ± 0.10
***OLE 3%***	3.01 ± 0.10	3.64 ± 0.08	4.47 ± 0.03	4.27 ± 0.08	4.95 ± 0.07	4.98 ± 0.09
***OLE 5%***	2.72 ± 0.09	3.38 ± 0.09	3.32 ± 0.06	3.11 ± 0.05	3.65 ± 0.04	3.81 ± 0.07
	***Coefficient of regression (R^2^)***					
***Control***	0.997 ± 0.001	0.968 ± 0.004	0.997 ± 0.002	0.996 ± 0.002	0.987 ± 0.002	0.989 ± 0.003
***OLE 1.5%***	0.997 ± 0.000	0.998 ± 0.000	0.994 ± 0.002	0.982 ± 0.004	0.995 ± 0.002	0.984 ± 0.004
***OLE 3%***	0.999 ± 0.001	0.993 ± 0.003	0.993 ± 0.001	0.966 ± 0.003	0.990 ± 0.001	0.963 ± 0.004
***OLE 5%***	0.999 ± 0.000	0.987 ± 0.002	0.990 ± 0.003	0.999 ± 0.000	0.993 ± 0.002	0.995 ± 0.001

**Table 4:** Viable counts of *Lactobacillus* sp. and *Streptococcus* sp. in yoghurt samples without (control) and with the addition of olive leaf extract (OLE 1.5%, OLE 3%, and OLE 5%) during 35 days of cold storage.

Storage Time (Days)	* Log CFU mL^−1^							
	*Lactobacillus* sp.				*Streptococcus* sp.			
	Control	OLE 1.5%	OLE 3%	OLE 5%	Control	OLE 1.5%	OLE 3%	OLE 5%
1	9.98 ± 1.05	9.68 ± 0.59	9.18 ± 1.38	7.65 ± 1.66	9.80 ± 0.94	10.27 ± 1.03	10.57 ± 1.03	9.87 ± 0.00
7	9.11 ± 0.68	9.05 ± 0.60	6.73 ± 1.30	6.97 ± 1.42	9.46 ± 0.81	10.17 ± 1.03	9.97 ± 1.06	8.97 ± 0.53
14	9.55 ± 0.75	7.22 ± 1.48	7.28 ± 1.50	6.85 ± 1.39	9.80 ± 0.99	9.09 ± 0.18	9.55 ± 0.50	9.36 ± 0.00
21	7.01 ± 1.43	7.09 ± 1.44	7.18 ± 1.47	7.22 ± 1.34	10.48 ± 1.35	9.33 ± 0.72	9.05 ± 0.15	9.02 ± 0.41
28	6.07 ± 1.25	7.50 ± 1.43	7.17 ± 1.32	5.99 ± 1.08	8.96 ± 0.40	9.81 ± 1.04	9.04 ± 0.00	9.21 ± 0.63
35	5.48 ± 0.79	5.63 ± 0.90	5.26 ± 0.88	5.08 ± 0.00	9.42 ± 0.58	8.89 ± 0.38	8.96 ± 0.41	8.92 ± 0.00

* Results are expressed as mean ± SD.

**Table 5:** Influence of extract content (%) and storage time on sensorial properties of yoghurts enriched with olive leaves extract.

Source of Variation	Appearance	Colour	Consistency	Odour	Syneresis	Taste	Total Score
Max. Score	1	1	4	2	2	10	20
**Extract content (%)**	*p* = 0.254	*p* = 0.045 *	*p* = 0.306	*p* = 0.001 *	*p* = 0.252	*p* = 0.001 *	*p* = 0.001 *
0	0.98 ± 0.00 ^a^	1.0 ± 0.00 ^b^	3.68 ± 0.08 ^a^	1.88 ± 0.04 ^b^	2.0 ± 0.00 ^a^	8.20 ± 0.20 ^b^	17.74 ± 0.02 ^b^
1.5	0.98 ± 0.00 ^a^	1.0 ± 0.00 ^b^	3.68 ± 0.08 ^a^	1.92 ± 0.04 ^b^	2.0 ± 0.00 ^a^	8.60 ± 0.20 ^b^	18.18 ± 0.02 ^b^
3	0.96 ± 0.02 ^a^	1.0 ± 0.00 ^b^	3.60 ± 0.08 ^a^	1.84 ± 0.04 ^ab^	1.96 ± 0.04 ^a^	8.00 ± 0.20 ^ab^	17.36 ± 0.03 ^ab^
5	0.96 ± 0.02 ^a^	0.96 ± 0.02 ^a^	3.60 ± 0.08 ^a^	1.76 ± 0.04 ^a^	2.0 ± 0.00 ^a^	7.40 ± 0.20 ^a^	16.68 ± 0.03 ^a^
**Storage time (day)**	*p* = 0.182	*p* = 0.067	*p* = 0.207	*p* = 0.022 *	*p* = 0.019 *	*p* = 0.001 *	*p* = 0.016 *
1	0.98 ± 0.02 ^a^	0.98 ± 0.02 ^a^	3.68 ± 0.08 ^a^	1.84 ± 0.04 ^ab^	2.0 ± 0.00 ^b^	8.40 ± 0.20 ^ab^	17.88 ± 0.03 ^ab^
7	0.98 ± 0.00 ^a^	1.0 ± 0.00 ^a^	3.68 ± 0.08 ^a^	1.84 ± 0.04 ^ab^	2.0 ± 0.00 ^b^	8.20 ± 0.20 ^ab^	17.70 ± 0.02 ^ab^
14	0.98 ± 0.00 ^a^	1.0 ± 0.00 ^a^	3.60 ± 0.08 ^a^	1.96 ± 0.00 ^b^	2.0 ± 0.00 ^b^	8.80 ± 0.20 ^b^	18.34 ± 0.02 ^b^
21	0.98 ± 0.00 ^a^	1.0 ± 0.00 ^a^	3.68 ± 0.08 ^a^	1.88 ± 0.04 ^ab^	2.0 ± 0.00 ^b^	8.40 ± 0.20 ^ab^	17.94 ± 0.02 ^ab^
28	0.92 ± 0.02 ^a^	0.98 ± 0.02 ^a^	3.52 ± 0.08 ^a^	1.80 ± 0.04 ^ab^	1.92 ± 0.04 ^a^	7.60 ± 0.40 ^ab^	16.74 ± 0.05 ^a^
35	0.96 ± 0.02 ^a^	0.98 ± 0.02 ^a^	3.68 ± 0.08 ^a^	1.72 ± 0.04 ^a^	2.0 ± 0.00 ^b^	7.00 ± 0.40 ^a^	16.34 ± 0.03 ^a^
**Grand mean**	0.96	0.98	3.60	1.84	2.00	8.0	17.38

* *p* ≤ 0.05. Results are expressed as mean ± SE. Values with different superscript letters within column are statistically different at *p* ≤ 0.05.

## Data Availability

Data is contained within the article.
